# Traditional Herbal Medicines, Bioactive Metabolites, and Plant Products Against COVID-19: Update on Clinical Trials and Mechanism of Actions

**DOI:** 10.3389/fphar.2021.671498

**Published:** 2021-05-28

**Authors:** Safaet Alam, Md. Moklesur Rahman Sarker, Sadia Afrin, Fahmida Tasnim Richi, Chao Zhao, Jin-Rong Zhou, Isa Naina Mohamed

**Affiliations:** ^1^ Department of Pharmacy, State University of Bangladesh, Dhaka, Bangladesh; ^2^ Pharmacology and Toxicology Research Division, Health Med Science Research Limited, Dhaka, Bangladesh; ^3^ Department of Pharmacy, Faculty of Pharmacy, University of Dhaka, Dhaka, Bangladesh; ^4^ College of Food Science, Fujian Agriculture and Forestry University, Fuzhou, China; ^5^ Nutrition/Metabolism Laboratory, Beth Israel Deaconess Medical Center, Harvard Medical School, Boston, MA, United States; ^6^ Pharmacology Department, Medical Faculty, Universiti Kebangsaan Malaysia (The National University of Malaysia), Kuala Lumpur, Malaysia

**Keywords:** anti-COVID-19, phytomedicine, antiviral, traditional Chinese medicine, Ayurved medicine, phytochemicals, anti-infective, bioactive metabolites

## Abstract

SARS-CoV-2 is the latest worldwide pandemic declared by the World Health Organization and there is no established anti-COVID-19 drug to combat this notorious situation except some recently approved vaccines. By affecting the global public health sector, this viral infection has created a disastrous situation associated with high morbidity and mortality rates along with remarkable cases of hospitalization because of its tendency to be high infective. These challenges forced researchers and leading pharmaceutical companies to find and develop cures for this novel strain of coronavirus. Besides, plants have a proven history of being notable wellsprings of potential drugs, including antiviral, antibacterial, and anticancer therapies. As a continuation of this approach, plant-based preparations and bioactive metabolites along with a notable number of traditional medicines, bioactive phytochemicals, traditional Chinese medicines, nutraceuticals, Ayurvedic preparations, and other plant-based products are being explored as possible therapeutics against COVID-19. Moreover, the unavailability of effective medicines against COVID-19 has driven researchers and members of the pharmaceutical, herbal, and related industries to conduct extensive investigations of plant-based products, especially those that have already shown antiviral properties. Even the recent invention of several vaccines has not eliminated doubts about safety and efficacy. As a consequence, many limited, unregulated clinical trials involving conventional mono- and poly-herbal therapies are being conducted in various areas of the world. Of the many clinical trials to establish such agents as credentialed sources of anti-COVID-19 medications, only a few have reached the landmark of completion. In this review, we have highlighted and focused on plant-based anti-COVID-19 clinical trials found in several scientific and authenticated databases. The aim is to allow researchers and innovators to identify promising and prospective anti-COVID-19 agents in clinical trials (either completed or recruiting) to establish them as novel therapies to address this unwanted pandemic.

## Introduction

**FIGURE 1:**
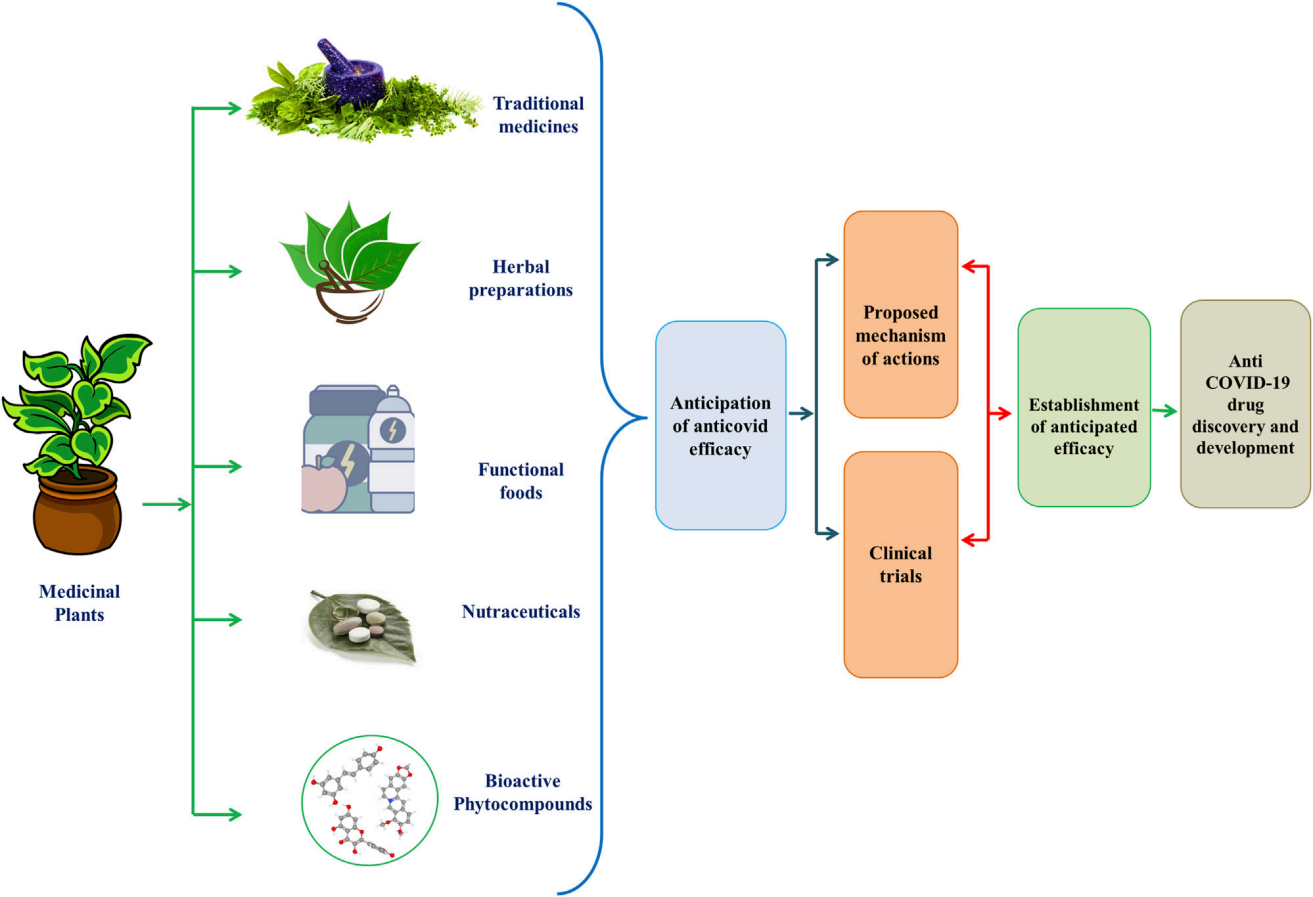
Graphical Abstract of Phytomedicines/Herbal Medicines/Bioactive compounds/Functional Foods/Nutraceuticals for the development of anti-COVID-19 therapies.

Coronavirus disease 2019 (COVID-19), caused by a newly identified strain of coronavirus, came under scrutiny in early December 2019 after an outbreak of pneumonia appeared in the city of Wuhan, Hubei Province, Central China (Abd El-Aziz and Stockand 2020; Li et al., 2020; Ludwig and Zarbock, 2020). Severe acute respiratory syndrome coronavirus (SARS-CoV) in 2002 and Middle East Respiratory Syndrome Corona Virus (MERS-CoV) in 2012 imposing critical menaces to humankind. Now, coronavirus has registered its third appearance in 2019 as a novel strain of coronavirus, SARS-CoV-2 which is extremely pathogenic and denominated as SARS-CoV-2 (Ludwig and Zarbock, 2020). SARS-CoV-2 has spread very quickly in several countries, with 109, 594, 835 confirmed cases of infection and about 2,424,060 deaths worldwide as of February 18, 2021, according to the WHO Coronavirus disease (COVID-19) Dashboard (Lai et al., 2020; Li et al., 2020; WHO, 2021). The WHO Director-General announced the COVID-19 epidemic to be a public health emergency of global significance on January 30, 2020. on February 11, 2020, the epidemic of the 2019-novel coronavirus disease (2019-nCoV) was given a new label. This was followed by the announcement of the COVID-19 outbreak as a global pandemic in March 2020 (Abd El-Aziz and Stockand, 2020; Guo et al., 2020; Ludwig and Zarbock, 2020). SARS-CoV-2 is a beta-coronavirus with enveloped and non-segmented positive-sense RNA (Guo et al., 2020). Like SARS-CoV, it uses the same receptor, angiotensin-converting enzyme 2 (ACE2) to infect humans, primarily through the respiratory system (Guo et al., 2020; Ludwig and Zarbock, 2020). Four essential structural proteins have been are identified as major drug targets in the virus: the glycoprotein of the surface spike (S), the membrane (M) protein, the glycoprotein of the small envelope (E), and the nucleocapsid (N) (Ludwig and Zarbock, 2020; Sarkar et al., 2020). Thus, finding effective and reliable therapeutic options for SARS-CoV-2 has become an unparalleled concern for researchers (Sarkar et al., 2020). Plant products may a wonderful gateway to the discovery and development of anti-COVID-19 therapeutics (Figure 1).

**FIGURE 2:**
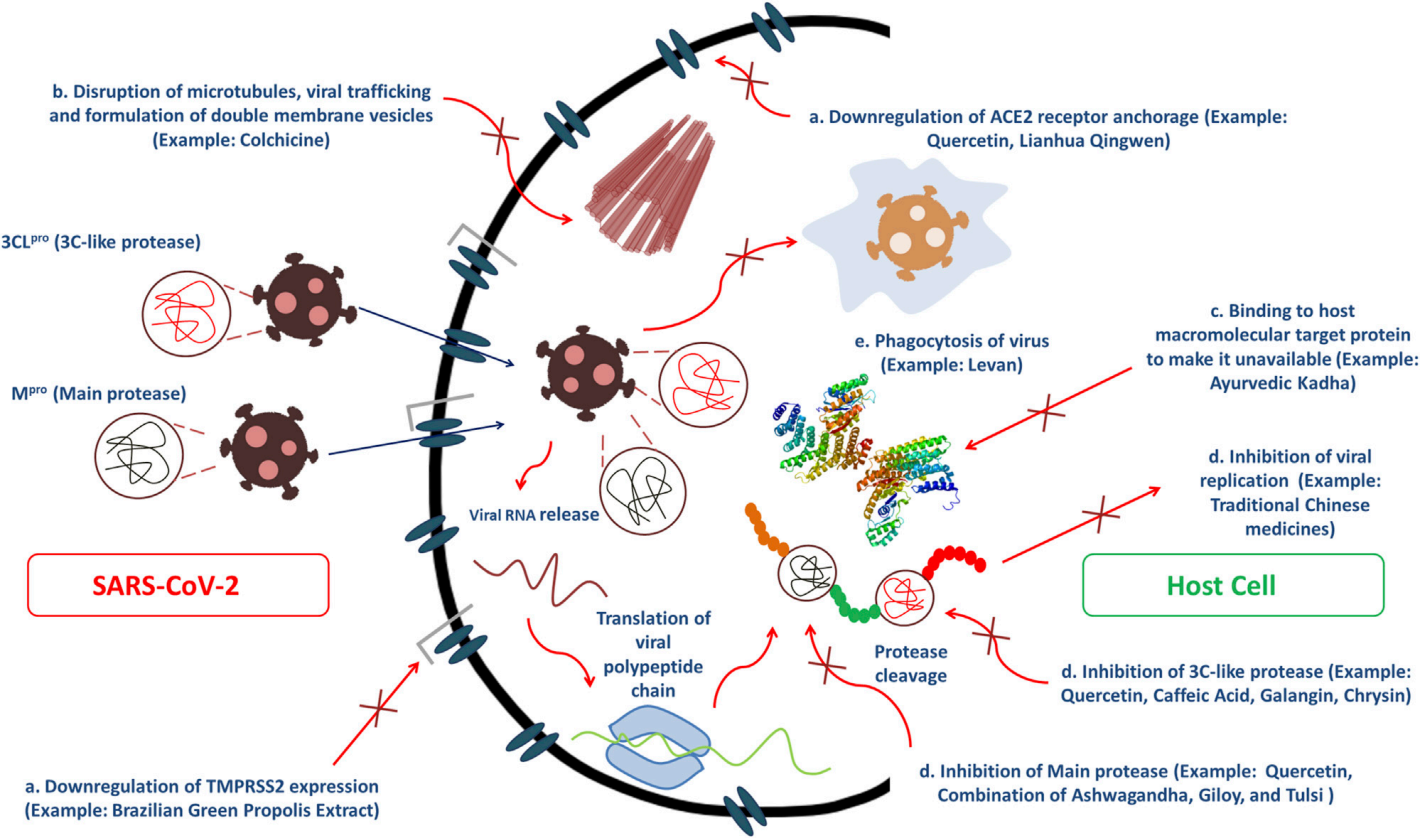
Several modes of bioactive phytoconstituents and traditional herbal medicines to exert anti-COVID-19 efficacy including **(A)** inhibition of main protease and 3C-like protease, **(B)** Disruption of microtubules, viral trafficking and formulation of double membrane vesicles, **(C)** Binding affinity toward host macromolecular target protein to make it unavailable and **(D)** Downregulation of ACE2 receptor anchorage and TMPRSS2 expression which ultimately causes inhibition of viral replication.

People across the world, especially from Asian regions like India, China and Japan, and some African nations have used plants as medicaments since the ancient era (Hoareau and DaSilva, 1999). Folkloric use of these plants among tribal people is mostly because of their profound availability and comparatively low cost (Jahan and Ahmet, 2020). Even in the modern era, plants are still very promising therapeutic sources to treat complications like pain, oxidative stress, cancer, diarrhea, depression, fever, and thrombosis, along with infectious diseases. This gives us hope that drugs can be developed to exert anti-COVID-19 efficacy from phyto wellsprings through several mechanism of actions (Figure 2) (Andrighetti-Fröhner et al., 2005; Alam et al., 2020; Jahan and Ahmet, 2020; Yang et al., 2020; Emon et al., 2021). The secondary metabolites alkaloids, flavonoids, polyphenols, tannins, lignins, coumarins, terpenoids, and stilbenes from medicinal plants are claimed to be efficacious in ameliorating infections from pathogenic microorganisms owing to its ability to arrest viral protein and enzymatic activities by binding with them, preventing viral penetration into and replication in the host cells (McCutcheon et al., 1995; Semple et al., 1998; Jahan and Ahmet, 2020). Consequently, several reports have suggested the possibility of inhibitory actions of plant-derived bioactive compounds against this novel strain of coronavirus, SARS-CoV-2 (Jahan and Ahmet, 2020).

Herbal medicines have also helped to mitigate the effects of contagious diseases like SARS-CoV. Evidence reinforces the view that herbal medicine may well be efficacious in managing and reducing the risk of COVID-19 as well. The National Health Commission of China has authorized the use of herbal medicine as an alternative remedy for COVID-19 in conjunction with Western medicine, and has released several recommendations on herbal therapy (Ang et al., 2020). Many doctors and researchers have indeed begun to use herbal medicines in human trials against SARS-CoV-2. Since many botanical drugs show antiviral efficacy, the use of herbal remedies for therapeutic purposes should not be underestimated. Currently, well-known herbal medicines with antiviral function are being used as supplementary treatments to suppress SARS-CoV-2 outbreaks, since conventional treatments are still not well flourished (Panyod et al., 2020). Traditional Chinese medicines (TCM) also have a rich history and many years of experience in the treatment and regulation of communicable diseases. They act by optimizing body immunity to pathogenic factors, balancing the immune response, reducing hyper-inflammatory states, and fostering body repair. Earlier TCM therapies have been found to prevent diseases from progressing to risky and critical conditions, which also has had efficacy in reducing the mortality rate. Giving new prospects for COVID-19 treatment, a set of current clinical findings have shown that TCM can play an important role in the treatment and prevention of COVID-19 (Ren et al., 2020). Ayurveda, which is of Indian origin, is another classical medical philosophy. It puts greater prominence on building body strength and the power of the mind. For respiratory disorders, different treatment options such as steam inhalation, immunomodulators, herbal infusions, and gargling hot water are used in Ayurveda, which also gives us hope in this pandemic situation (Golechha, 2020).

In the absence of specific evidence-based therapy against SARS-CoV-2, some researchers have shifted toward plant-based therapies, as many drugs are plant materials or their derivatives (Silveira et al., 2020). Despite the development of vaccines in recent days, extensive multi-site clinical studies are still required to ensure actual efficacy and safety (Silveira et al., 2020). Thus, notable importance is given to the discovery of potential anti-COVID-19 natural products and herbal medicines as records of plant-based therapeutics displayed promising efficacy against several viruses by strengthening the immune system (Huang J. et al., 2020; Zhang et al., 2020). Thus, in this study, we report on clinical studies with completed and/or recruiting status that are exploring the efficacy and safety of traditional medicines, isolated compounds, functional foods, nutraceuticals, herbal preparations, and other plant-based products like those used in Chinese medicines and Ayurved, used against SARS-CoV-2. The aim of those studies is to facilitate the use of such materials as remedial and preventive therapies against COVID-19 and to draw the attention of drug developers in this field while new synthetic drugs are still under investigation and have not provided satisfactory results (Panyod et al., 2020).

### Article Search Strategy and Methodology

**FIGURE 3:**
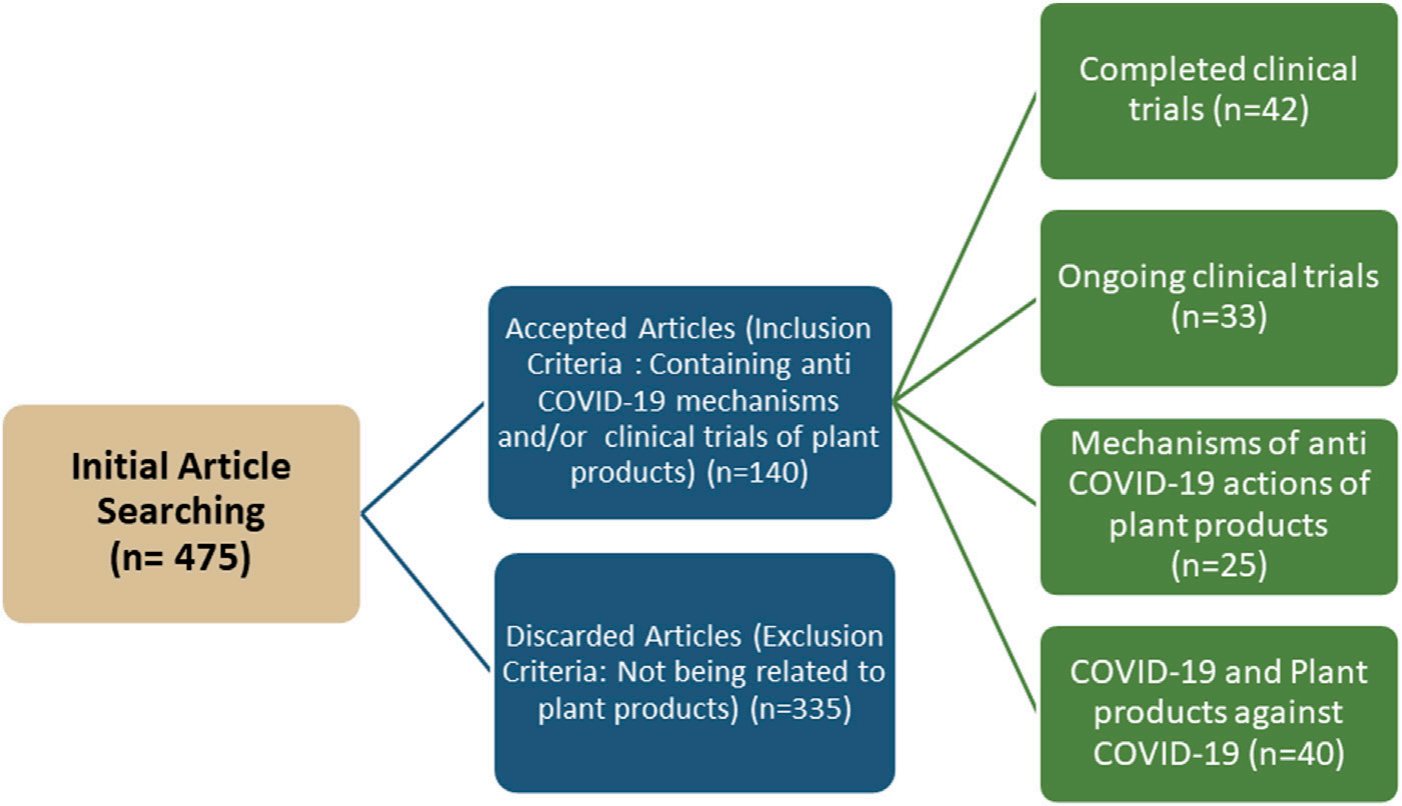
Flowchart for article search, screening and selection in literature review.

To summarize the findings regarding plant-based products and traditional medicines with completed and/or recruiting clinical trial status; a literature search was conducted using PubMed, Web of Science, Scopus, ScienceDirect, Wiley Online Library, Google Scholar, and CNKI Scholar databases. There was also a search for clinical trials on the ClinicalTrials.gov website (https://www.clinicaltrials.gov/) because of its authenticity. The keywords used in the searches included5 “COVID-19,” “SARS-CoV-2,” “phytomedicines,” “bioactive compounds,” “Ayurved medicine,” “traditional Chinese medicine,” “herbal medicine,” “anti-COVID-19,” “plant-based medicine” and “clinical trials”. Only articles reporting completed and/or recruiting clinical studies and/or mechanisms of actions were included in the final review. There were 475 papers and clinical trials initially identified. After eliminating titles and abstracts related to clinical trials and/or mechanisms of action, 140 unique articles and clinical trials were identified that met the criteria and purpose of this review (Figure 3).

### Completed Clinical Trials of Phyto-Based Promising Preparations (Bioactive Metabolites/Traditional Medicines/Plant Extracts/Nutraceuticals/Functional Foods/Chinese Medicines/Ayurved Medicines/Plant Based Other Preparations)

#### Clinical Studies of Bioactive Metabolites to Treat COVID-19

##### Colchicine

Colchicine is a lipid-soluble, tricyclic alkaloid with bioavailability that ranges from 24 to 88% and a long terminal half-life ranging from 20 to 40 h (Slobodnick et al., 2015). While the therapeutic activities of colchicine have been known for centuries, the medication was first approved by the United States Food and Drug Administration (FDA) in 2009 (Slobodnick et al., 2015). Even before FDA approval, researchers have worked to expand the spectrum of clinical uses of colchicine from gout and Mediterranean family fever (Slobodnick et al., 2015). Besides, colchicine is also being tested against COVID-19 infection because of its ability to interfere with the inflammatory immune response. For instance, it inhibits the chemotaxis of inflammatory leukocytes particularly neutrophils and monocytes. Then it also compromises their adhesion to endothelial cells by inhibiting the expression of the adhesion molecule E-selectin, consequently preventing them from migrating to inflamed tissue. The plant metabolites have also been associated with a reduction in the production of superoxide free radicals, a decline in tumor necrosis factor and the deactivation of the NLRP3 inflammasome (Montealegre-Gómez et al., 2020; Schlesinger et al., 2020). In particular, colchicine tends to reduce the release of cytokines to manage inflammatory activity (Di Lorenzo et al., 2020).

By arresting SARS-CoV-2 replication through disrupting microtubules, which are climacteric for viral trafficking and the formation of double-membrane vesicles, colchicine could be efficacious against SARS-CoV-2 infection (Perricone et al., 2020). Colchicine is a microtubule disassembling agent since it inhibits the polymerization of tubulin protein, which has a deleterious effect on microtubule polymerization (Montealegre-Gómez et al., 2020). When colchicine binds to β-tubulin subunit due to its strong affinity, the αβ-tubulin heterodimeric subunits lose their straight conformation, culminating in curved tubulin heterodimers as a consequence of which a microtubule polymer with a different morphology emerges (Montealegre-Gómez et al., 2020; Schlesinger et al., 2020). As the cytoskeleton's most essential element is microtubules, colchicine interferes with several cellular functions which are greatly conditioned upon cytoskeleton (Montealegre-Gómez et al., 2020). It explains the antiviral efficacy of colchicine as some viruses, particularly Coronaviruses, rely on microtubules and the cytoskeleton for their entry into the cell, replication and transcription of the viral genome. As per hypothesis colchicine can prevent coronavirus from entering the cell because entry requires the spike protein to interact with cytoskeletal proteins, specifically tubulin. Colchicine can further interfere with coronavirus replication, since microtubules are critical for the development of double-membrane vesicles in affected cells, as well as the transfer and assembly of spike proteins into virions, all of which are key steps in the viral replication (Schlesinger et al., 2020).

The use of 0.5 mg of colchicine per day during the early infection stage (phase 1) of COVID-19 is appropriate because the host has sufficient immunity and it will prevent the disease from progressing to phase 2 and/or 3. But in the pulmonary phase (phase 2), it would be reasonable to use 0.5 mg of colchicine (bid) as the patient's immune system has become weakened. The most critical phase is 3, which is characterized by cytokine storms that culminate in a systemic hyper inflammatory condition that can lead to multiple organ failure and ultimately, death in the patient. Attempting to control Cytokine Storms would be feasible with 0.5 mg of colchicine (qd or bid) in monotherapy or with glucocorticoids (dexamethasone) as a combination therapy (Vitiello et al., 2021).

A phase 3 randomized, double-blind, placebo-controlled clinical trial was conducted on colchicine to evaluate its candidacy for COVID-19 treatment. The triple masking (participant, care provider, and investigator) approach was coordinated by the Montreal Heart Institute (MHI) in Canada, South Africa, South America, Europe, and the United States of America. Initially, 6,000 patients who were COVID-19 positive in terms of nasopharyngeal PCR test were recruited in the clinical trial for the assessment of the effectiveness of colchicine compared to placebo (colchicine group: placebo group allocation ratio 1:1; half patients would receive colchicine while the rest half would receive placebo) for one month (NCT04322682, 2020; Montreal Heart Institute, 2020). From the recruitment, 4506 COVID-19 patients finally participated in the trial. Colchicine was given to patients (*n* = 2,253) at risk of complications as soon as the diagnosis of SARS-CoV-2 infection was confirmed by PCR (at the initial stage of COVID-19), which lowered the risk of development of a severe form of the disease followed by abatement of the incidence of hospitalizations compared to placebo (*n* = 2,253). In this contact-less COLCORONA trial (ClinicalTrials.gov Identifier: NCT04322682), patients infected with SARS-CoV-2 participated who were at least 40 years old and of either sex. They had been diagnosed with COVID-19 infection within the last day. Each patient in the treatment group received 0.5 mg colchicine orally twice daily as a primary treatment; for the first 3 days, followed by one dose daily for the next 27 days. Similarly, the placebo group received placebo tablets at the same dose and same pattern like the colchicine group. Patients who were hospitalized or under emergency consideration of being hospitalized were kept out of this trial. The trial was conducted only among patients who were staying at home (NCT04322682, 2020; Montreal Heart Institute, 2020). The outcomes revealed that colchicine alleviated the risk of death and hospitalization 21% more in the treatment group than in the placebo group. Another promising outcome of this statistically significant clinical trial was a decline in hospitalizations of 25%, the use of mechanical ventilation by 50%, and mortality by 44% among 4,159 patients who were tested positive for corona infection in nasopharyngeal PCR tests. These outcomes were accredited to colchicine as a promising oral drug for treating coronavirus-infected non-hospitalized patients (Montreal Heart Institute, 2020).

##### Quercetin

Quercetin comes from the Latin word “Quercetum” meaning oak forest. It belongs to the class of flavonols, and it cannot be formed in the human psyche (Lakhanpal and Rai, 2007). Quercetin is chemically 3,3′,4′5,7-pentahydroxyflavone. It is widely available in many fruits, leaves, seeds, and grains, though it generally couples with residual sugars to form quercetin glycosides (Li et al., 2016). Researchers have found that supplementation with quercetin can facilitate anti-inflammatory, antioxidant, anti-viral, and immuno-protective activities (Uchide and Toyoda, 2011;Colunga Biancatelli et al., 2020). Because of its auspicious antiviral efficacy in inhibiting polymerases, proteases, and reverse transcriptase; suppressing DNA gyrase; and binding viral capsid proteins, quercetin has been subjected to many models of viral infection (Debiaggi et al., 1990; Cushine and Lamb, 2005). Theoretically, Quercetin can be a prominent antiviral agent as it inhibits 3-chymotrypsin-like protease (3CLpro) and papain-like protease (PLpro), which are the main druggable targets of SARS-CoV-2. In addition, quercetin has been one of the best performing ligands for the S protein: ACE2 receptor interface in structure-based drug discovery. As a consequence, it can restrict viral identification at the host cell surface and/or interrupt host-virus interactions at the receptor level and its subsequent entry into the cell (Derosa et al., 2020).

A randomized clinical trial was conducted from March 20, 2020, to July 31, 2020, on quercetin to evaluate its effectiveness (both prophylaxis and treatment) against SARS-CoV-2 as a primary treatment. It was an open-label masking approach that was coordinated for 3 months. In this randomized trial (ClinicalTrials.gov Identifier: NCT04377789) 447 participants were categorized as a no intervention (no quercetin group), a quercetin prophylaxis group, and a quercetin treatment group. Those in the quercetin prophylaxis group with no history of COVID-19 infection were given 500 mg of quercetin daily. The members of the quercetin treatment group had confirmed cases of COVID-19 and they were provided with 1,000 mg quercetin daily. Clinical data of the prevalence of COVID-19, standardized mortality rates, and morbidity rates were collected for further analysis as major aims (NCT04377789, 2020). Although the aim of this clinical trial was to evaluate the pervasiveness of quercetin in prophylaxis group and the mortality rate in treatment group (James et al., 2020), to the best of our knowledge no conspicuous outcome has been published so far in any online databases (Saeedi-Boroujeni and Mahmoudian-Sani, 2021).

#### Clinical Studies of Traditional Chinese medicine (TCM) to Treat COVID-19

##### Lianhua Qingwen

Lianhua Qingwen (LHQW), a traditional Chinese medicine, is now officially registered in the 2015 edition of Chinese Pharmacopeia (Li et al., 2020; Ling et al., 2020). The LHQW preparation is available as granules, capsule and decoction dosage forms, and was prepared by using 13 components: dried fruit of *Forsythia suspensa* (Thunb.) Vahl (known as ‘lian- qiao’ in China) [Oleaceae; Forsythiae fructus], dried stem or aerial part of *Ephedra sinica* Stapf from Ephedraceae family (honey-fried Herba Ephedrae which is locally known as ‘zhi-ma-huang’), dried flower buds of *Lonicera japonica* Thunb. (locally known as ‘jin-yin-hua) [Caprifoliaceae; Lonicerae Japonicae Flos], dried root of *Isatis indigotica* Fortune ex Lindl. (Chinese name ‘ban-lan-gen’) [Brassicaceae; Isatidis Radix], kernel of *Prunus arminiaca* L. (locally known as ‘chao-ku-xing-ren’) [Rosaceae; Semen Armeniacae Amarum], dried aerial part of *Pogostemon cablin* (Blanco) Benth. (locally known as ‘guang-huo-xiang’) [Labiatae; Pogostemonis Herba], dried rhizome and remnants of leaf stems of *Dtyopteris crassirhiaoma* Nakai (locally known as ‘mian-ma-guanzhong’ [Dryopteridaceae; Dryopteridis Crassirhizomatis Rhizoma], aerial part of *Houttuynia cordata* Thunb. (locally known as ‘yu-xing-cao’) [Saururaceae; Houttuyniae Herba], dried roots and rhizomes of *Rheum palmatum* L., *Rheum tanguticum* Maxim. ex Balf. and *Rheum officinale* Baill (locally known as ‘da-huang’) [Polygonaceae; Rhei Radix et Rhizoma], dried root and rhizome of *Glycyrrhiza uralensis* Fisch. (locally known as ‘gan-cao’) [Fabaceae; Glycyrrhizae Radix et Rhizoma], root and rhizome of *Rhodiola crenulata* (Hook. f. et Thoms.) H. Ohba (locally known as ‘hong-jing-tian’) [Crassulaceae; Rhodiolae Crenulatae Radix et Rhizoma], mineral agent Gypsum Fibrosum (known as ‘shi-gao’), and L-menthol (known as ‘bo-he-nao’) (Chen et al., 2021; Li et al., 2021).

It displays a wide spectrum of antiviral functions, primarily because of its immunological modulation and inhibitory impact on the replication of the virus, and its inhibitory effect on the release of pro-inflammatory cytokines. Evidence suggests that LHQW has a therapeutic effect on COVID-19 because of its strong binding capacity with M^pro^ and ACE2; which are SARS-CoV-2’s therapeutic targets. Thus, it can be proved to be beneficial for treating COVID-19 as a supplementary and synergetic strategy (Li L.-C. et al., 2020; Ling et al., 2020). Twelve representative components were the key constituents of LHQW, and they were evaluated as chemical markers: salidroside, chlorogenic acid, Forsythoside E, crypto-chlorogenic acid, amygdalin, sweroside, hyperin, rutin, Forsythoside A, phillyrin, rhein, and glycyrrhizic acid (Li et al., 2020; Ling et al., 2020). However, a very latest study has been conducted to establish the cause responsible for SARS-CoV-2 inhibition and core basic anti-COVID-19 mechanism of pharmacologically active ingredients of LHQW by Prof. Caisheng Wu and Prof. Yifeng Chai from the School of Pharmacy, Xiamen University. In accordance with their research work, the base of the action of LHQW against COVID-19 resides in pernicious interplay of ACE2 targeted LHQW components with ACE2 and spike (S) protein complex. Basically, by scrutinizing ACE2 bio-chromatographic stationary phase, which was drawn out via analyzing human plasma and urine after multiple administration of LHQW on the basis of HRMS and intelligent non-targeted data mining techniques, 85 components were found. Among them, only some components namely, Glycyrrhizic acid, Amygdalin, Forsythoside A, Forsythoside I, Rhein, Aloe-emodin, Neochlorogenic acid and Prunasin were manifested to have good binding affinity to ACE2 and ultimately the ACE2 inhibition was carried out by forsythoside A, forsythoside I, rhein, neochlorogenic acid and its isomers. Furthermore, computer-aided molecular docking analysis also gave proof that, these components effectively bind with the contact surface of ACE2 and S protein complex; hence, the complex is affected. And it is suspected to be the main reason for LHQW to inhibit SARS-CoV-2 (Chen et al., 2021).

Chinese medicine LHQW and its role as an adjuvant therapy in COVID-19 therapy were clinically analyzed in a retrospective study conducted from January 11 to January 30, 2020. The results were posted in the Chinese Journal of Experimental Formulas. The study was conducted on 21 members of a treatment group who received conventional treatment combined with LHQW granules, 1 bag at a time, 3 times a day. During the trial, the treatment group showed a statistically significant difference (*p* < 0.05) in the rates at which shortness of breath, sputum, fever, and cough symptoms disappeared. In addition, the duration of fever in the treatment group was 1.5 days shorter than in the control group (Kaitao et al., 2020).

In another study, clinical data of 103 patients from January 1 to January 27, 2020, were collected. The treatment and control groups had 63 and 38 patients, respectively. In the treatment group, symptomatic therapy, conventional treatment (intervention to support nutrition), antiviral and antibacterial drug treatment were paired with LHQW granules for 10-days. LHQW granules were used as adjuvant treatment in this case. The control group received conventional therapy only. The treatment group was significantly better (*p* < 0.05) in terms of the rate of disappearance of fever, cough and fatigue, shortness of breath, and downward trend. However, between the two groups, there was no statistically significant discrepancy in the median duration of fever (Ruibing et al., 2020).

##### Toujie Quwen

This traditional Chinese medicine may exert antioxidant, antiviral, and anti-inflammatory effects, together with modulating the immune system. Thus, it plays a primary protective role in the lungs, through certain possible bio targets of COVID-19. Toujie Quwen (TJQW) works on EGFR (Estimated Glomerular Filtration Rate), CASP3 (Caspase 3), STAT3 (Signal transducer and activator of transcription 3), ESR1 (Estrogen Receptor 1), FPR2 (Formyl-peptide receptor-2), BCL2L1 (Bcl-2-like protein 1), BDKRB2 (B2 bradykinin receptor), MPO (Myeloperoxidase), and ACE (Angiotensin-converting enzyme) which have been identified as possible therapeutic and core pharmacological targets, most of which have been reported to be implicated in pulmonary inflammation (Huang J. et al., 2020).

Toujie Quwen (TJQW) has been formulated with a focus on utilizing heat-clearing and detoxifying botanical drugs to reduce heat and phlegm, to relax the body parts, to stabilize and retain critical energy, and to remove pathogenic factors along with spleen invigoration and dampness elimination (Huang Y. et al., 2020). TJQW contains 16 components of TCM: dried fruit of *Forsythia suspensa* (Thunb.) Vahl (known as ‘lian- qiao’ in China) [Oleaceae; Forsythiae fructus], edible tulip which is locally known as ‘shan-ci-gu’ (Tulipa gesneriana L. from Liliaceae family), *Lonicera japonica* Thunb. (Japanese honeysuckle flower, jin-yin-hua) from Caprifoliaceae family, dried root of the medicinal plant *Scutellariae baicalensis* Georgi (locally known as ‘huang-qin’) [Lamiaceae; Radix Scutellariae baicalensis], leaves of *Isatis indigotica* Fortune ex Lindl. (Chinese name ‘da-qing-ye’) [Brassicaceae; Folium Isatidis], dried roots of *Bupleurum chinense* DC. (known as ‘chai-hu’) [Apiaceae; Bupleurum Radix], *Artemisia apiacea* Hance (known as ‘qing-hao’) from Compositae family, the cast-off shell of the *Cryptotympana pustulata* Fabricius (known as ‘chan-tui’) [Cicadidae; Periostracum Cicadae], dried roots of *Peucedanum praeruptorum* Dunn (known as ‘qian-hu’) [Apiaceae; Radix Peucedani], *Fritillaria cirrhosa* D.Don (known as ‘chuan-bei-mu’) from Liliaceae family, *Fritillaria thunbergii* Miq. (known as ‘zhe-bei-mu’) also from Liliaceae family, dried sclerotium of *Wolfiporia cocos* (F.A. Wolf) Ryvarden & Gilb. (an edible medicinal mushroom known as ‘fu-ling’) [Polyporaceae; Poria cocos], unripe fruit of *Prunus mume* (Siebold) Siebold & Zucc.) (known as ‘wu-mei’) [Rosaceae; Fructus Mume], dried root of *Scrophularia ningpoensis* Hemsl. (known as ‘xuan-shen’) [Scrophulariaceae; Radix Scrophulariae], *Astragalus propinquus* Schischkin (known as ‘huang-qi’) from Fabaceae family, and root of *Pseudostellaria heterophylla* (Miq.) Pax (known as ‘tai-zi-shen’) [Caryophyllaceae; Radix Pseudostellariae] (Huang J. et al., 2020). The bioactive metabolites in TJQW, such as umbelliprenin, quercetin, kaempferol, luteolin, praeruptorin E, stigmasterol, and oroxylin A, have possible anti-inflammatory and antiviral activities and may be effective against SARS-CoV-2 infection (Huang Y. et al., 2020).

A clinical trial was conducted to evaluate the effectiveness of TJQW granules as an adjuvant therapy in combination with Arbidol against SARS-CoV-2 and the impact on the expression of specific T cells. A total of 73 patients diagnosed with COVID-19 were randomly divided into the treatment group (37 cases) and control group (36 cases). Upregulation of the lymphocyte count and downregulation of C-reactive protein (CRP) and a reduced TCM syndrome score were observed in the treatment group. The discrepancy in results between the treatment and control group was statistically significant (*p* < 0.05). In contrast to the control group, there was a statistically significant increase in the number of CD4+/CD8+ cells in the treatment group (Xiaoxia et al., 2020).

##### Shufeng Jiedu

In traditional Chinese medicine, Shufeng Jiedu capsule (SFJDC), is often used to cure influenza, and is now suggested for the management of COVID-19 infections (Chen et al., 2020). SFJDC is a popular formulation prepared from—the root and rhizome of *Polygonum cuspidatum* Sieb. et Zucc (known as ‘hu-zhang’) [Polygonaceae; Polygoni Cuspidati Rhizoma et Radix], dried fruit of *Forsythia suspensa* (Thunb.) Vahl (known as ‘lian-qiao’) [Oleaceae; Forsythiae Fructus], root of *Isatis indigotica* Fort. (known as ‘ban-lan-gen’) [Brassicaceae; Isatidis Radix], dried root of *Bupleurum chinense* DC. (known as ‘chai-hu’) [Apiaceae; Bupleuri Radix], Herba Patriniae which is scientifically known as *Patrinia scabiosaefolia* Link and locally known as ‘bai-jiang-cao’ from Caprifoliaceae family, ‘ma-bian-cao’ (*Verbena officinalis* L. from Verbenaceae family), dried roots of *Phragmites communis* Trinius (locally known as ‘lu-gen’) [Poaceae; Phragmitis Rhizoma], and dried root and rhizome of *Glycyrrhiza uralensis* Fisch. (locally known as ‘gan-cao’) [Fabaceae; Glycyrrhizae Radix et Rhizoma] (Chen et al., 2020).

To study the effectiveness of SFJDC as an adjuvant therapy together with Arbidol to treat uncomplicated COVID-19, 200 patients were divided into an observation group and a control group of 100 each (Qi et al., 2020). For two weeks, the observation group was given 2.08 g of Shufeng Jiedu capsule and 0.2 g Arbidol three times a day while the control group was given only 0.2 g Arbidol (tid) (Liu et al., 2020; Qi et al., 2020; Sun et al., 2020). Compared to the control group, the chest CT condition, the white blood cell count, and lymphocytes percentage were significantly improved in the observation group (*p* < 0.05). The fevers in the observation group were shorter than in the control group. Also, the observation group’s overall success rate was 88%, compared to 75% for the control group (Qi et al., 2020).

Clinical data for 70 patients who were clinically diagnosed with COVID-19 in Bozhou People’s Hospital and who received care from January 31, 2020, to February 11, 2020, were compiled and analyzed to determine the efficacy of SFJDC as an adjuvant therapy together with Arbidol Hydrochloride to treat COVID-19 (Qi et al., 2020). Thirty patients received only 0.2 g Arbidol hydrochloride capsules, and the other 40 patients received the combination of two drugs: 2.08 g of Shufeng Jiedu capsule and 0.2 g Arbidol three times a day (Liu et al., 2020; Qi et al., 2020; Sun et al., 2020). The negative conversion time was slightly shorter for the group that received both drugs compared to the other group. Also, the latter group had a statistically significant difference in antipyretic duration, dry cough disappearance period, nasal congestion, runny nose, pharyngeal distress, fatigue, diarrhea, and negative conversion time for the novel coronavirus compared to the former group (*p* < 0.05) (Qu et al., 2020).

##### Qingfei Touxie Fuzheng Recipe

Qingfei Touxie Fuzheng is a traditional Chinese medicine. Studies suggest that using this recipe in conjunction with Western medicine was more successful for treating COVID-19 than using Western medicine alone. The likely mechanism is the upregulation of antiviral factors and the downregulation of pro-inflammatory factors (Ding et al., 2020).

To examine the therapeutic effectiveness of the Qingfei Touxie Fuzheng recipe as an adjuvant therapy to treat COVID-19, clinical data on 100 patients were collected (Ding et al., 2020). The 51 patients in the treatment group received a course of antiviral and anti-infection-based Western medicine namely recombinant human interferon α1b for injection (5 million U, bid) or ribavirin (0.5 g, bid) along with the Qingfei Touxie Fuzheng recipe (150 mL, bid) and the control group only received aforementioned western medicine (Ding et al., 2020; Liu et al., 2020; Sun et al., 2020). The levels of ESR, CRP, and IL-6 were substantially reduced in the treatment group relative to the control group, and the discrepancy was statistically significant (*p* < 0.05). Also, in alleviating clinical manifestations of fever, cough, expectoration, chest tightness, and shortness of breath; facilitating the absorption of pulmonary lesions; and improving oxygenation, the results for the treatment group were considerably better than those for the control group (Ding et al., 2020).

##### Other Traditional Chinese Formulations

The following clinical trials were completed, and data were collected from the literature and/or ClinicalTrials.gov but the names of the products were not available:

Clinical data of 67 patients were obtained to examine the effectiveness of combining traditional Chinese and Western medicine in the treatment of non-critical COVID-19 patients receiving traditional Chinese as adjuvant therapy. Of the 67 patients, 18 were in the Western medicine group, and 49 were in the integrated traditional Chinese and Western medicine group (Jia et al., 2020). The former group was given oxygen therapy, antiviral drugs, anti-inflammatory drugs, immunologic agents and other treatment to alleviate symptoms and the latter group was provided TCM decoction and Chinese patent medicine namely Shufeng Jiedu capsule, Lianhua Qingwen granule etc along with aforementioned western medicine (Jia et al., 2020; Ni et al., 2020; Wang et al., 2021). However, some critically ill patients were given anti-inflammatory medicine for immune regulation and supporting therapy of gamma globulin. The latter group was given a decoction of Chinese medicine. In terms of clinical results, the overall trajectory of the disease, antipyretic timeframe, and rate of CT improvement was not statistically different (*p* < 0.05). The latter group’s dampness toxin stagnation syndrome and heat toxin blocking lung syndrome were 65.31% and 34.69% respectively. In addition, their stays in the hospital were shorter and they had fewer severe effects. No transformation from mild to chronically ill state occurred in either group during the surveillance period (Jia et al., 2020).

Clinical data from January 15 to February 8, 2020, on 52 patients with COVID-19 were obtained to examine the therapeutic efficacy of combining Chinese and Western medicine to treat SARS-CoV-2 infection in patients receiving conventional Chinese medicine as adjuvant therapy where among the 52 patients, 23 were male and 29 were female (Xia et al., 2020). The Western medicine group was given arbidol, ribavirin, alpha interferon, lopinavir/ritonavir, oseltamivir for treating viral infections as well as to prevent or treat any other viral or secondary infections moxifloxacin, levofloxacin, azithromycin, cephalosporins penicillin drugs were provided. Gamma globulin and methylprednisolone was delivered as a supplementary treatment regime, while the group treated with both Chinese and Western medicine was also given Lianhua Qingwen Granules, Ganlu Xiaodu Dan, Huoxiang Zhengqi Water, different Decoction of Shidu Yufei, Yudu Biaofei, NIaxing Shigan and varying injection of Xuebijing, Phlegm Heat Qing, Shengmai, Shenfu etc (Xia et al., 2020; An et al., 2021). For the latter group, the rate at which accompanying symptoms disappeared and the clinical cure rates were 85.3% and 91.2% respectively. In addition, the duration of clinical symptoms, mean hospital stay, time to return to normal body temperature, and score on the TCM syndrome scale for the latter group were statistically significantly lower compared to the former group (Xia et al., 2020).

In another study, 19 confirmed patients were treated with a combination of traditional Chinese (as an adjuvant) and Western medicine (antiviral and oxygen therapy) and it was reported that the fever, cough, exhaustion, and other symptoms improved substantially following treatment (Zhongqian et al., 2020; Zhou et al., 2020). Hospitalization lasted 16.36 ± 4.95 days, no patient became extremely ill, and the rate of effectiveness of the combination approaches was 100% (Zhongqian et al., 2020).

According to Ruibing et al., 2020, another clinical study was conducted on 103 patients’ data were collected between January 1 and January 27, 2020, to determine the efficacy of integrated Chinese and western medicine in patients who received LHQW granules as adjuvant (Ruibing et al., 2020). The treatment group had 63 patients, and the control group had 38. In the former group, symptomatic therapy and medicine to relieve cough and asthma, conventional treatment (intervention to support nutrition), glucocorticoid and antiviral, antibacterial, immunomodulatory drug treatment were paired with LHQW granules for 10-days (Ruibing et al., 2020; Zhou et al., 2020). The latter group received previously-mentioned conventional therapy only. The treatment group was significantly better in the disappearance rates for fever, cough, and fatigue, and shortness of breath had a downward trend (*p* < 0.05). However, the difference in the median duration of fever was not statistically significant (Ruibing et al., 2020).

In another study, to evaluate the efficacy of conventional Chinese medicine against SARS-CoV-2 as primary therapy, 30 patients diagnosed with COVID-19 in February 2020 were arbitrarily categorized into three groups of 10 cases each. Group A was the placebo group; Group B was handled with conventional Chinese medicine and fumigation; and Group C received traditional Chinese medicine along with fumigation and absorption in conjunction with a rich level of vitamin C. In groups B and C, the degree of recovery from the disease state and the elimination of weakness, coughing, sore throat, and chest tightness were better than in group A. Moreover, the outcomes for group C were better than those of group B (Wang et al., 2020).

Based on a clinical study by Tan et al. (2020), clinical data on 50 hospitalized patients with confirmed and suspected cases of COVID-19 were obtained to examine the therapeutic efficacy of traditional Chinese medicine as an adjuvant therapy in treating pneumonia from the novel coronavirus (Tan et al., 2020). They received discrete TCM syndrome differentiation therapy (Chinese decoction along with antiviral drugs, anti-inflammatory agents, oxygen therapy and glucocorticoid) (Tan et al., 2020; Zhou et al., 2020) and their rates for being clinically cured, effective, or totally effective were 46.00, 52.00, and 98.00% respectively. In addition, fever, sweating, fatigue, discomfort in the body, tightness in the chest, shortness of breath, and other signs also diminished at a greater pace (Tan et al., 2020).

In another study, clinical data on 103 patients from January 27, 2020, to March 1, 2020, were gathered. Of the 103 patients, 51 were in the treatment group and 52 were in the control group. The former group was given a combination of traditional Chinese and Western medicine with the former acting as an adjuvant. The latter group received conventional treatment (antiviral, and anti-inflammatory drugs, oxygen therapy, and glucocorticoid) (Meng et al., 2020; Zhou et al., 2020). Compared to the control group, the absolute value of lymphocytes, albumin content, number of cases of enhanced lung absorption, and cure rate improved significantly, while CRP and the mortality rate declined substantially in the treatment group (Meng et al., 2020).

Besides, in another study, clinical data on 23 hospitalized COVID-19 patients who were 23–72 years old were examined to determine the therapeutic efficacy of a combination of traditional Chinese and Western medicine (antiviral and anti-inflammatory drugs, oxygen therapy and glucocorticoid) with the former acting as an adjuvant to treat SARS-CoV-2. Of the 23 patients, 10 were male and 13 were female (Zhou et al., 2020; Zhu et al., 2020). Clinical symptoms of fever, sputum, chest tightness, and asthma digestive tract symptoms were recorded as 78.3, 26.1, 26.1, and 8.7% respectively. Hematological tests revealed that the overall number of white blood cells was normal. However, 16 patients reported low lymphocytes, and two patients were observed to have an irregular liver function. Triglycerides and cholesterol levels were elevated and CRP was reduced at discharge. Chest CT scans confirmed that there were several shades of ground glass under the pleura. The condition was exacerbated by interstitial thickening. Antiviral, anti-infective, and oxygen inhalation therapies were used to cure all cases. Non-invasive ventilation was provided to only one critical patient. After obtaining the negative results of the nucleic acid screening, all patients were released (Zhu et al., 2020).

These trials of patented traditional Chinese medicines were conducted to ensure safety and efficacy in treating SARS-CoV-2 infection, although the exact formulations or recipes are not publicly available.

#### Clinical Studies of Ayurvedic Medicine to Treat COVID-19

##### Ayurvedic Kadha

Ayurvedic medicine and its extracts have been used for a very long time in the prevention and cure of viral maladies. Ayurvedic Kadha is prepared from using several Indian botanical drugs including ‘tulsi’ (*Ocimum sanctum* L. from Lamiaceae family), ‘haldi’ (*Curcuma longa* L. from Zingiberaceae family), ‘giloy’ (*Tinospora cordifolia* (Lour.) Merr. from Menispermaceae family), ‘black pepper’ (*Piper nigrum* L. belonging to Piperaceae family), ‘ginger’ (*Zingiber officinale* Roscoe from Zingiberaceae family), ‘clove’ (*Syzygium aromaticum* (L.) Merr. & L.M.Perry from Myrtaceae family), ‘cardamom’ (*Elettaria cardamomum* (L.) Maton from Zingiberaceae family), ‘lemon’ (*Citrus limon* (L.) Osbeck from Rutaceae family) and ‘ashwagandha’ (*Withania somnifera* (L.) Dunal from Solanaceae family) (Maurya and Sharma, 2020). Preparing Kadha (decoction) for oral intake is a significant Ayurvedic procedure for amplifying the effect of pharmacological agents in botanical drugs. Kadha is an ancient type of medicine made by combining botanical drugs and spices. The Indian government suggested the use of Kadha during the COVID-19 epidemic to enhance immunity and promote healing (Maurya and Sharma, 2020). The Ayurvedic Kadha-based phytochemicals have a strong binding affinity with the various viral and host macromolecular target proteins. This suggests that they can exert antiviral efficacy by regulating viral replication and proliferation in host cells. (Maurya and Sharma, 2020).

An observational study (ClinicalTrials.gov Identifier: NCT04387643) with 52 participants was conducted for the 30 days between March 1, 2020, and April 2, 2020. The aim was to assess the efficacy of Ayurvedic kadha as a primary therapy to protect healthcare workers during the COVID-19 pandemic. After the study period, their physical and psychological health, their ability to cope with distress, and their capacity for self-help were evaluated (NCT04387643, 2020).

##### Guduchi Ghan Vati

*Tinospora cordifolia* (Lour.) Merr., locally known as “Guduchi” or “Giloe” is a large, deciduous, and glabrous climbing shrub from the Menispermaceae family. It is available throughout the tropical regions in India and China, and it has a notable history in folkloric use (Upadhyay et al., 2010; Patgiri et al., 2014). An aqueous extract of *T. cordifolia* is used to prepare the popular Ayurvedic formulation, Guduchi GhanVati, which is listed in the Ayurvedic Pharmacopoeia of India. With a wide spectrum of pharmacological activities, Guduchi Ghan Vati, traditional Indian medicine, is generally prescribed as an immunomodulatory and antioxidant agent (Upadhyay et al., 2010; Patgiri et al., 2014). In recent studies, Guduchi GhanVati was also found to be effective against SARS-CoV-2 (Kumar et al., 2020a; Kumar et al., 2020b).

Clinical data from May 12 to June 15, 2020, was collected on 91 participants between 18 and 75 years of age. The aim was to evaluate the efficacy of Ayurveda (Guduchi GhanVati-extract of *Tinospora cordifolia*) as a primary therapy to manage confirmed COVID-19 patients who were asymptomatic to mildly symptomatic. In this trial (ClinicalTrials.gov: Identifier: NCT04480398), participants were divided into two groups. The Ayurvedic group comprised 40 individuals who were given two tablets (500 mg each) of Guduchi Ghan Vati (extract of *Tinospora cordifolia*) orally twice each day after a meal for 28 days. The control group of 51 individuals was provided with conventional and standard therapy (NCT04480398, 2020). The results showed a reduction of 11.7% in symptoms in the control group after an average of 1.8 days and none developed any exaggerated symptoms in the Ayurveda group. The Ayurvedic group’s virologic clearance at day 7 and at day 14 was 97.5% and 100% respectively, while for the control group it was 15.6% and 82.3% respectively. The differences were statistically significant. The average stay in the hospital for the Ayurvedic group was 6.4 days; for the control group, it was 12.8 days (Kumar et al., 2020a; Kumar et al., 2020b).

An open-label single-arm feasibility trial was conducted to assess the efficacy of Guduchi Ghan Vati (an aqueous extract of *Tinospora cordifolia*) as a primary therapy to manage COVID-19 infection in 46 asymptomatic patients. Only 40 patients completed the 14-days trial period. All the patients were provided with two tablets (1,000 mg) 2 times a day for 14 days, but there was no control group. After the treatments, no one showed COVID-19 symptoms. Viral clearance was 32.5% and 95%, on day 3 and day 7, respectively. Data for day 14 revealed all respondents’ test reports were negative (Kumar et al., 2020a; Kumar et al., 2020b).

##### Combination of Ashwagandha, Giloy, and Tulsi

*Withania somnifera* (L.) Dunal from Solanaceae family (locally known as ‘ashwagandha’), *Tinospora cordifolia* (Lour.) Merr. from Menispermaceae family (locally known as ‘giloy’) and *Ocimum sanctum* L. from Lamiaceae family (locally known as ‘tulsi’) are notable herbal plants used in the Ayurvedic treatment system. All these immunomodulator plants have displayed promising immunity-boosting efficacy against infection, which has drawn the attention of researchers to assess their anti-COVID-19 activity (Shree et al., 2020). Bioactive metabolites of Ashwagandha, Giloy, and Tulsi were predicted to show prominent efficacy against SARS-CoV-2 infection in recent studies. An in-silico study revealed that this formulation can arrest the main protease (Mpro or 3Clpro) of a novel strain of coronavirus because of phytochemicals like somniferin, isorientin 4′-O-glucoside 2″-O-p-hydroxybenzoate, withanoside V, tinocordiside, somniferine, and vicenin. ADME/T profiling also showed its safety and drug-like activity (Shree et al., 2020).

A community-based participatory study was conducted to evaluate the effectiveness of Ayurveda intervention as supportive care for patients with mild to moderate COVID-19. Patients with severe cases were excluded from this study. In this open-label approach (ClinicalTrials.gov Identifier: NCT04716647), 28 people with confirmed infections with SARS-CoV-2 participated from October 9, 2020, to December 18, 2020. As primary therapy, they were given oral tablets of Ashwagandha, Giloy, and Tulsi. Based on age, weight, and the severity of symptoms, patients were given between 250 mg and 5 g of Ashwagandha, between 500 mg and 1 g of Giloy, and between 500 mg and 1 g of Tulsi. Then clinical recovery time, the number of patients whose nasopharyngeal swab test converted to negative in the study period, and other clinical outcome data were gathered for evaluation (NCT04716647, 2020). The gain from Ayurveda was better for relief of symptoms, so cline recovery within seven days was proposed. Although there was no control group, a statistically significant disparity was found by data triangulation from different normal treatments. The Ayurvedic procedure can have a positive impact, particularly for people suffering from mild to moderate symptoms of COVID-19 (Kulkarni et al., 2021).

##### Tinospora cordifolia and Piper longum

*Tinospora cordifolia* (Lour.) Merr. from Menispermaceae family is a plant found in regions of India and China. In traditional Ayurvedic medicine, it is used in the formulation of Guduchi Ghan Vati (Upadhyay et al., 2010; Patgiri et al., 2014). It can also be coadministered with *Piper longum* L. (from Piperaceae family)*,* another popular medicinal plant used in the Ayurvedic system. *P. longum* is locally known as “Pippali” and “Indian Long Pepper.” Pippali is a traditional Ayurvedic complementary ingredient that enhances the bioavailability and absorption of the other active components, and *P. longum* itself possesses notable antiviral activity (Pandey et al., 2013; Priya and Kumari, 2017).

A placebo-controlled, randomized trial (ClinicalTrials.gov Identifier: NCT04621903) was conducted from October 8 to October 27, 2020, to evaluate the efficiency and safety of an Ayurvedic combination (Guduchi and Pipli) as a primary treatment for 28 asymptomatic to mildly symptomatic patients who were confirmed patients of COVID-19. The participants were given Guduchi (*Tinospora Cordifolia*; 300 mg) and Pipli (*Piper Longum* 75 mg) twice daily (NCT04621903, 2020). By day 3 after treatment, 71.1% of the patients in the treatment group recovered, and 50% of the placebo group recovered. By day 7, 100% of patients in the treatment group recovered, but only 60% of the control group recovered. The recovery response rate in the treatment group was higher than the control group. The probability of delayed healing process from infection in the treatment group was significantly decreased by 40% (Devpura et al., 2021).

##### Other Ayurvedic Formulations Without Products/Ingredients Named

An open-label clinical trial (ClinicalTrials.gov Identifier: NCT04345549) was conducted on Ayurveda as a primary therapy to evaluate its efficacy as supportive care in self-management for flu-like symptoms of COVID-19 for people in self-isolation. In total 18 patients participated from February 26, 2020, to March 30, 2020. People with known hypersensitivity to any Ayurveda botanical drugs were excluded from the study. All the participants were instructed to self-isolate for 7 days and to practice grooming and self-care according to the prescribed directives. Participants were also recommended to use plants, lifestyle, and yoga to implement Ayurveda therapy. In line with Ayurveda, individuals were recommended to gargle with warm water, practice steam inhalation, take paracetamol to control body temperature, and drink plenty of fluids with ginger, lemon, turmeric, or honey, and relax both body and mind. They were also advised to practice yoga breathing. After the treatment, data on the duration of fever and the score for the severity of influenza symptoms were collected and analyzed to determine the efficacy of this approach as supportive care (NCT04345549, 2020).

A non-randomized single-blinded trial (ClinicalTrials.gov Identifier: NCT04351542) was conducted on Ayurveda as a primary therapy and supportive care for flu-like illness during the COVID-19 pandemic. This trial started on March 6, 2020, with 32 participants and ended primarily on April 6, 2020. People were divided into an Ayurveda care group and an active comparator (usual care) group. Participants from the former group were given individualized Ayurveda treatment, and usual care was recommended for the latter group. After the study, the duration of the fever, the score for the severity of symptoms, and reported improvement of patients were observed and further analyzed and compared to determine the suitability of Ayurveda as supportive care for flu-like symptoms during COVID-19 (NCT04351542, 2020).

The Ayurvedic medicine was conducted in the hope of ensuring safety and efficacy against COVID-19 infection, but the precise intervention was not disclosed by the respective researcher groups.

#### Clinical Studies of Herbal Medicine and Functional Foods to Treat COVID-19

##### Fuzheng Huayu Capsule/Tablet

The Fuzheng Huayu capsule or tablet is a patented Chinese herbal medicine formulated after decades of laboratory and clinical study by the Institute of Liver disease, Shanghai University of Traditional Chinese Medicine. The Shanghai Modern Pharmaceutical Company developed composite capsules that are prescribed for treating a wide spectrum of hepatic disorders (Shi et al., 2020; Wu et al., 2020). The herbal extraction combines six Chinese herbal medicines: fruits of *Schisandra chinensis* (Turcz.) Baill. (known as ‘wu-wei-zi’) [Schisandraceae; Fructus Schisandra chinensis], *Paecilomyces hepiali* Chen & Dai from Trichocomaceae family (locally known as ‘chong-cao’), root and rhizome of *Salviae miltiorrhizae* Bunge (Chinese name ‘dan-shen’) [Lamiaceae; Salviae Miltiorrhizae Radix et Rhizoma], *Pinus armandii* Franch. from Pinaceae family (known as ‘song-hua-feng’), seed of *Prunus persica* (L.) Batsch (known as ‘tao-ren’) [Rosaceae; Semen Persicae], and *Gynostemma pentaphyllum* (Thunb.) Makino from Cucurbitaceae family (known as ‘jiao-gu-lan’) (Shi et al., 2020; Wu et al., 2020). In treating chronic liver disorders, an FZHY capsule can ameliorate the complications caused by viral hepatitis B and C, which gives a ray of hope about its antiviral efficacy (Chen et al., 2019).

A phase 4 case-controlled, non-randomized, open-label clinical trial was conducted from February 1, 2020, to April 15, 2020, to assess the impact of Fuzheng Huayu as a primary therapy on lung inflammation in 66 patients with severe SARS-CoV-2 pneumonia and to diminish the advancing rate to severe type. In this trial (ClinicalTrials.gov Identifier: NCT04645407), 66 persons were divided into two groups: those that received Fuzheng Huayu and the control group. For 14 days, those in the experimental group were given Fuzheng Huayu tablets 0.4 g/tablet, 1.6 g/time, 3 times/day after meals, in addition to conventional therapy. The control group was given only conventional therapy. The patients were monitored for improvement in chest CT, remission or progression of critical illness, the clinical remission of respiratory symptoms. They were given routine blood tests as well as tests of CRP level, procalcitonin level, and oxygen saturation, and the data were analyzed to find primary and secondary outcomes (NCT04645407, 2020).

##### Cretan IAMA

Since the Bronze Age, the island of Crete has been noted for its use of herbal medicine. Previous studies have revealed the antioxidant function of certain plants from Crete. In addition, recent research on Cretan herbal wellsprings done by the University of Crete in collaboration with the University of Leiden, Netherlands, has led to a new formula (Lionis, 2015). Olvos Science SA has recently introduced an over-the-counter drug in Greece produced as soft-gel capsules (Lionis, 2015). “Cretan IAMA” is a combination of essential oils extracted from three aromatic botanical drugs from Lamiaceae family: Coridοthymus capitatus (*Thymus capitatus* (L.) Hoffmanns. & Link, *Salvia fruticosa* Mill., and *Origanum dictamnus* L. in a fixed ratio and dissolved in extra virgin oil. This formulation acts synergistically to promote the natural increase in the body’s defense mechanism (Lionis, 2015).

A phase 3, single-arm, open-label small interventional proof-of-concept (POC) study (ClinicalTrials.gov Identifier: NCT04705753) was conducted on Cretan IAMA (CAPeo) to test its therapeutic efficacy as a primary treatment for resolving the frequency and duration of symptoms and their intensity in patients. The study was conducted from April 1, 2020, to October 15, 2020. Twenty participants received Cretan IAMA soft gels at 1 ml per day as a dietary supplement (NCT04705753, 2020).

##### Nigella sativa and Honey

*Nigella sativa* L. is a popular herbal plant with ethnomedicinal importance from the Ranunculaceae family. It is commonly known as “Black Cumin” or by its local name, “Kalojira” (Forouzanfar et al., 2014). *N. sativa* has been traditionally used for over 2000 years in Middle Eastern folklore medicines and traditional Arabian herbal medicines (Forouzanfar et al., 2014). In previous studies, researchers discovered its anti-COVID-19 efficacy by decreasing SARS-CoV replication in cell cultures (Ulasli et al., 2014). Moreover, an *in silico* study revealed that notable compounds like thymoquinone α-hederin and nigelledine possess a greater affinity for multiple SARS-CoV-2 enzymes and proteins. The energy complex scores of those compounds are even higher than those for hydroxychloroquine, chloroquine, and Favipiravir, which have shown some anti-SARS-CoV-2 efficacy (Ashraf et al., 2020). Besides, because of the overlapping pharmacological profiles of honey and *N. sativa*, it is believed that this combination can reduce the severity of disease and control viral replication. Honey mixed with *N. sativa* has already shown remarkable potential for treating several other diseases. This gives researchers a ray of hope to use this combination in the current pandemic (Ameen et al., 2011; Bhatti et al., 2016; Moghimipour et al., 2019)

*Bacillus subtilis,* isolated from honey, produces a very promising antiviral component known as levan (*β*-2,6-fructan) that functions against many pathogenic respiratory RNA viruses. By facilitating the aggregation of cells and viruses, levan amplifies the phagocytosis procedure. Another proposed mechanism of antiviral activity in honey is through the nitric oxide pathway. Studies have shown that honey raised the concentration of NO at the cellular level. In turn, this neurotransmitter shuts off viral protease enzymes by blocking their capability for viral polyprotein cleavage. As a consequence, viral RNA synthesis is occluded at a very early stage. Then, viral replication, the accumulation of viral protein, and the release of the virus from infected cells are hampered sequentially. Whether NO can express antiviral activity against SARS-CoV-2 or simply prevent the progression of the disease is still uncertain. Caffeic acid, phenethyl ester (CAPE), galangin, and chrysin are natural phenolic chemical compounds from honey. They have a strong ability to inhibit the viral 3-chymotrypsin-like cysteine protease (3CLpro), the enzyme responsible for the cleavage of the viral polyprotein pp1a and pp1ab. Then, nonstructural proteins (nsps) and RNA-dependent RNA polymerase (RdRp) cannot form as pp1a and pp1ab. The polyprotein cleavage process is interrupted, which ensures that replication of structural protein RNA; viral RNA synthesis, and virion assembly do not occur. In that way, SARS-CoV-2 replication will eventually be halted (Al-Hatamleh et al., 2020).

The molecular docking system also found that nigellidine and α-hederin of *N. sativa* might be active SARS-CoV-2 inhibitor compounds because they exhibit the most binding affinity with SARS-CoV-2 molecular targets 6LU7 and 2GTB, which are protease and peptidase respectively. They might also induce an antiviral cellular response by raising the number of CD4 cells (Ulasli et al., 2014; Koshak and Koshak, 2020).

A phase 3 randomized, placebo-controlled, open-label, add-on, cohort, adaptive, investigator-initiated interventional trial was conducted from April 30, 2020, to August 30, 2020 on honey and *Nigella sativa* to evaluate their role as a co-therapy in treating COVID-19. A triple masking approach ensures that the care provider, the investigator, and the outcomes assessor do not know who is receiving the drug and who is receiving a placebo. In this study (ClinicalTrials.gov Identifier: NCT04347382), 313 confirmed COVID-19 patients were divided into an experimental group and a placebo comparator group (who received standard medical care). The former group was given 80 mg per kg per day of *N. sativa* seed as powder which was ground and put into a capsule and natural honey 1 gm per kg per day for a maximum of 14 days. The latter group was given an empty capsule with 250 ml of distilled water and conventional therapy, which includes supportive medical care and antibacterial or antiviral if advised. After the treatment, clinical data for the two groups were collected and analyzed. They included the number of days between the last positive result for a COVID-19 PCR test and a negative result, the severity of symptom progression, the duration of hospital stays, clinical grade status, the 30-days mortality rate, duration of fever, and oxygen saturation at room air (NCT04347382, 2020). A lower clinical score was observed in the honey and nigella group. By day 6, 63.6% of patients with moderate COVID-19 infection resumed their normal activity, while only 28% of severe cases could do so. Alleviation of disease symptoms, substantially earlier viral clearance, and a considerable reduction in mortality among severe patients were observed in the honey and nigella group compared to the control group. No adverse effects from honey and Nigella sativa were observed in the treatment group (Ashraf et al., 2020)

##### Brazilian Green Propolis Extract (EPP-AF)

Propolis is a resinous component from plant exudates created by honey bees. It’s been used in traditional herbal medicine since ancient times and is frequently used as a supplement for the immune system and healthcare. A few categories of propolis, such as Brazilian green propolis, are thoroughly appreciated for their therapeutic potential. Propolis is produced primarily by bees from substances that they gather from particular plants, such as *Baccharis dracunculifolia* DC. from Asteraceae family (Berretta et al., 2020).

Depending on the plant species in each location, the formulation of propolis differs. Some of the most typical propolis constituents are myricetin, caffeic acid phenethyl ester, hesperetin and pinocembrin, hesperetin, limonin, quercetin, and kaempferol (Berretta et al., 2020). The extract of propolis and some of its components help to reduce viral replication. They also exert inhibitory effects on the signaling pathways of ACE2, TMPRSS2, and PAK1 that are very relevant targets of the SARS-CoV-2. By reducing TMPRSS2 expression and ACE2 anchorage, propolis hampered the entry of the virus into the cell (Berretta et al., 2020). In the understanding of the existing urgency triggered by the COVID-19 pandemic and insufficient therapeutic possibilities, propolis was proposed as an effective, convenient, and possibly relevant therapeutic alternative, easily accessible as an organic nutrient and dietary supplement (Berretta et al., 2020).

A phase 3, randomized, open, pilot clinical study (ClinicalTrials.gov Identifier: NCT04480593) was conducted on Brazilian green propolis extract (EPP-AF) with hospitalized COVID-19 patients. It ran from June 2, 2020, to August 30, 2020, and its aim was to evaluate the candidacy of this extract as an adjuvant therapy option along with standard treatment including antibiotics or antivirals, vasopressor support, corticosteroids etc. against SARS-CoV-2 infection. In this single (outcomes-assessor) masking approach, 120 patients were randomly separated into three categories. The control group received standard conventional treatment. The first experimental group received 400 mg per day green propolis extract (EPP-AF) and conventional treatment. A second experimental group received 800 mg per day EPP-AF and conventional treatment. After the treatment, oxygen therapy dependency time, hospitalization time, the occurrence of side effects, the rate and severity of acute kidney injury, the need for renal replacement therapy, the need for vasopressor and ICU, invasive oxygenation time, plasma CRP variation data were collected from all three groups, analyzed, and compared (NCT04480593, 2020).

### Ongoing Clinical Trials of Promising Phyto-Based Preparations (Bioactive Metabolites, Traditional Medicines, Plant Extracts, Nutraceuticals, Functional Foods, Chinese Medicines, Ayurved Medicines, And Other Plant-Based Preparations)

There are trials of several promising and potent phyto-based formulations to treat SARS-CoV-2 infections, which are currently in the recruiting phase or in different stages. It is anticipated that these formulations will be potential sources in the discovery and development of anti-COVID-19 medicaments. This is because of their previous history of antiviral activities. Some of the promising formulations/agents are presented in Table 1.

**TABLE 1:** Ongoing clinical trials of several plants, functional foods, and plant based products including bioactive phytocompounds, traditional medicines, nutraceuticals and similar other preparations against SARS-CoV-2 infection.

No	Drugs	Therapy type	Phase	Participant numbers	Intervention	Outcomes	ClinicalTrials. Gov Identifier	References
1	Fuzheng Huayu Tablet	Primary therapy	Phase II	160 participants	0.4 g/tablet, 1.6 g/time, 3 times/day	The improvement proportion of pulmonary fibrosis, Blood oxygen saturation, Clinical symptom score, The 6-min walk distance	NCT04279197	NCT04279197 (2020)
2	Natural honey	Adjuvant therapy	Phase III	1,000 participants	Natural honey supplement 1 gm/kg/day divided into 2–3 doses for 14 days	Rate of recovery from positive to negative swaps, fever to normal temperature in days, Resolution of lung inflammation in CT or X ray, 30 days mortality rate, number of days till reaching negative swab results	NCT04323345	NCT04323345 (2020)
3	Anluohuaxian	Primary therapy	Not applicable	750 participants	Six grams each time, twice a day	Changes in high-resolution computer tomography of the lung, Change in 6-min walking distance, Changes in vital capacity of the lung	NCT04334265	NCT04334265 (2020)
4	Escin	Adjuvant therapy	Phase II, Phase III	120 participants	Oral admisntration of standard therapy and Escin tablet for 12 days (40 mg thrice a day)	Determination of mortality rate, the differences in oxygen intake methods, time of hospitalization (days), time of hospitalization in intensive care units, pulmonary function	NCT04322344	NCT04322344 (2020)
5	*Caesalpinia spinosa* (Molina) Kuntze extract (P2Et)	Adjuvant therapy	Phase II, Phase III	100 participants	P2Et active extract capsule equivalent to 250 mg of P2Et every 12 h for 14 days + Standard care	The efficacy of P2Et in reducing the length of hospital stay of patients with clinical suspicion or confirmed case of COVID-19	NCT04410510	NCT04410510 (2020)
	(Fabaceae)							
6	*Nigella sativa* L. (Ranunculaceae)	Primary therapy	Phase II	200 participants	Nigella sativa	Determination of proportion of patients who are clinically recovered, normalization of chest radiograph, rate of complications	NCT04401202	NCT04401202 (2020)
					Black seed oil in 500 mg capsules			
7	Essential oil	Primary therapy	Not applicable	65 participants	Essential oil Blend	Determination of State Trait Anxiety Scale (STAI-S) at 15 min	NCT04495842	NCT04495842 (2020)
					5 drops of on a tester strip			
8	Plant polyphenol	Primary therapy	Phase II	200 participants	Both plant polyphenols and placebo is introduced individually along with vitamin D3 10,000 IU	Reduction rate of hospitalization at 21 days from enrollment	NCT04400890	NCT04400890 (2020)
9	Silymarin	Adjuvant therapy	Phase III	50 participants	Silymarin oral 420 mg/day in 3 divided doses	Time to clinical improvement, clinical outcome, duration of mechanical ventilation, hospitalization, virologic response	NCT04394208	NCT04394208 (2020)
10	ArtemiC (curcumin, artemisinin, vitamin C, and Boswellia serrata)	Adjuvant therapy	Phase II	50 participants	ArtemiC will be sprayed orally twice a day for the first 2 days in the treatment period	Time to clinical improvement, Time to negative COVID-19 PCR	NCT04382040	NCT04382040 (2020)
11	Medicinal cannabis (*Cannabis sativa* L. from Cannabaceae family)	Primary therapy	Phase II	200,000 participants	Cannabis, medical	Treatment of COVID-19, treatment of symptoms	NCT03944447	NCT03944447 (2020)
12	Jing-Guan-Fang (JGF)	Primary therapy	Not applicable	300 participants	Jing-Guan-Fang (JGF)	The number of COVID-19 patients after this preventive treatment	NCT04388644	NCT04388644 (2020)
13	Licorice extract	Adjuvant therapy	Not applicable	70 participants	Licorice capsules; 250 mg standardized extract (25% Glycyrrhizin—62.5 mg) for 10 days	Increased number of people recovering from COVID-19	NCT04487964	NCT04487964 (2020)
14	Iota-Carrageenan	Primary therapy	Phase IV	400 participants	A nasal spray with Iota-Carrageenan or placebo 4 times a day	Progression to a more severe disease state, defined as need for oxygen therapy, lasting of disease, incidence of COVID-19 disease onset in the first week after treatment	NCT04521322	NCT04521322 (2020)
15	Acai palm berry extract (*Euterpe oleracea* Mart. from Arecaceae family)	Primary therapy	Phase II	480 participants	One capsule (520 mg) of Açaí Palm Berry every 8 h for a total of 3 capsules a day for 30 days	7-point ordinal symptom scale, need for mechanical ventilation, need for hospitalization	NCT04404218	NCT04404218 (2020)
16	QuadraMune™ (Composed of four natural ingredients)	Primary therapy	Not applicable	500 participants	Two pills of QuadraMune (TM) daily for 12 weeks	Prevention of COVID-19	NCT04421391	NCT04421391 (2020)
17	Phenolic monoterpenes + colchicine	Adjuvant therapy	Phase II	200 participants	Colchicine along with phenolic monoterpenes added to standard treatment in patients with COVID-19 infection	Improvement in clinical, radiological and laboratory manifestations will be estimated in treated group compared to control one	NCT04392141	NCT04392141 (2020)
18	Cannabidol	Primary therapy	Phase I, Phase II	400 participants	Oral administration of Cannabidiol (150 mg twice daily) for 14 days	Evaluation of the impact of Cannabidol on the cytokine profile with severe and critically COVID-19 infected people along with safety and efficacy profile	NCT04731116	NCT04731116 (2020)
19	Resistant	Primary therapy	Phase II, Phase III	1,500 participants	Twenty grams for 14 days in a twice daily pattern where nonresistant starch was used in the same amount as placebo	Determination of hospitalization rate for COVID-19 associated complications	NCT04342689	NCT04342689 (2020)
	Starch							
20	Colchicine	Adjuvant therapy	Phase III	102 participants	An preliminary dose of 1.5 mg followed by 0.5 mg twice daily during the next 7 days and 0.5 mg once daily until the completion of 14 days treatment	Assessment of changes in the patients' clinical status through the 7 points ordinal scale WHO R&D Blueprint expert group along with IL-6 concentrations	NCT04667780	NCT04667780 (2020)
		Primary therapy	Phase II	70 participants	Initial dose of 1.2 mg followed by 0.6 mg after 2 h on day 1. After that 0.6 mg of two doses up to 14th day	Assessment of decreased risk of progression into ARDS requiring upraised oxygen needs, mechanical ventilation and mortality	NCT04363437	NCT04363437 (2020)
21	Special Chinese medicine	Primary therapy	Not applicable	150 participants	Lung and spleen qi deficiency syndrome: 9 g French pinellia (*Pinellia ternata* (Thunb.) Makino from Araceae family), 10 g chenpi (*Citrus aurantium* L. from Rutaceae family), 15 g Codonopsis (*Codonopsis pilosula* (Franch.) Nannf. from Campanulaceae family), 30 g sunburn astragalus (*Astragalus membranaceus* (Fisch.) Bunge from Fabaceae family), 6 g *Amomum villosum* Lour. from Zingiberaceae family, and Licorice 6 g etc.	Assessment of changes in CM diagnostic pattern & clinical characteristics along with body constitution scores	NCT04544605	NCT04544605 (2020)
					Qi and Yin deficiency syndrome: root of *Salviae miltiorrhizae* Bunge 10 g, [Lamiaceae; North and south radix salviae], 15 g *Ophiopogon japonicus* (Thunb.) Ker Gawl. from Asparagaceae family, 6 g American ginseng (*Panax quinquefolius* L. from family Araliaceae family), 6 g Schisandra (*Schisandra chinensis* (Turcz.) Baill. from Schisandraceae family), 6 g gypsum l5 g, 10 g light bamboo leaves (*Bambusa vulgaris* Schrad. from Poaceae family), 10 g mulberry leaves (*Morus alba* L. from Moraceae family), 15 g reed root (*Ceanothus americanus* L. from Ramnaceae family), 15 g *Salvia miltiorrhiza* Bunge from Lamiaceae family, 6 g raw liquorice etc.			
22	Nicotine	Primary therapy	Phase III	1,633 participants	3.5 mg: day 1 to day 3	Determination of COVID-19 seroconversion between week o and week 19 after randomization	NCT04583410	NCT04583410 (2020)
					Seven milligrams: day 4 to day 9			
					10.5 mg: day 10 to day 15 14 mg: day 16 to day 98			
					10.5 mg: Day 99 to day 105			
					Seven milligrams: day 106 to day 112			
					3.5 mg: day 113 to day 119			
					As Nicotine patch			
		Primary therapy	Phase III	220 participants	0.5 patch for day 1 and day 2, 1 patch for day 3 and day 4, 1.5 patches for day 5 and day 6 where 2 patches from day 7 to the day discharge from hospital where one patch contains 7 mg nicotine	Determination of any he unfavorable outcome on day 14	NCT04608201	NCT04608201 (2020)
		Primary therapy	Phase III	220 participants	Two patches of 7 mg/day Treatment at 14 mg/day during mechanical ventilation since after first successful extubation followed by dose decrement	Determination of inhibition of the penetration and propagation of SARS-CoV2 by nicotine	NCT04598594	NCT04598594 (2020)
23	Hesperidin	Primary therapy	Phase II	216 participants	Capsules containing 0.5 gm of hesperidin in the evening and at bed time with water	Determination of proportion of subjects with COVID-19 symptoms	NCT04715932	NCT04715932 (2020)
24	Resveratrol + Zinc	Primary therapy	Phase II	60 participants	2 grams of resveratrol twice a day + Zinc picolinate 50 mg for thrice a day for 5 days	Assessment of reduction of COVID-19 viral load and its severity	NCT04542993	NCT04542993 (2020)
25	Melatonin	Primary therapy	Phase II	30 participants	Ten milligrams thrice a day dose day for 14 days	Determination of cumulative incidence of treatment-emergent adverse effects	NCT04474483	NCT04474483 (2020)
		Adjuvant therapy	Not applicable	55 participants	Nine milligrams dose of melatonin for seven to ten nights	Determination of modulation of immune system	NCT04409522	NCT04409522 (2020)
		Primary therapy	Phase II	18 participants	Maximum daily dose 500 mg per day	Determination of impact of Melatonin on mortality rate and hospital stay	NCT04568863	NCT04568863 (2020)
		Primary therapy	Not applicable	150 participants	Ten milligrams melatonin at bedtime	Electronically tracking of symptom severity	NCT04530539	NCT04530539 (2020)
		Primary therapy	Phase II, Phase III	450 participants	Two milligrams of prolonged release melatonin orally before bedtime for 12 weeks	Determination of prophylaxis efficacy of melatonin	NCT04353128	NCT04353128 (2020)

## Conclusion

Assessment of plant-based preparations including traditional medicines, bioactive compounds, and functional foods or nutraceuticals to battle the novel strain of coronavirus could provide a gigantic triumph for the distorted public health system. This expectation is based on the tremendous contributions that metabolites or herbal preparations have made in the past to address many kinds of serious ailments including infectious diseases. In this article, we have presented and discussed the data from several completed clinical trials that may yield valuable new treatments for COVID-19. They may be independent therapies or complementary or alternative medicines to manage COVID-19. Many prospective metabolites and plant-based herbal preparations in different forms of delivery led to promising outcomes in preclinical studies, and they are in various stages of clinical trials. However, to evaluate the effectiveness and safety profiles of these phytochemicals and mono- and poly-herbal formulations, it is crucial to perform extensive and more rigorous, high-quality, and evidence-based medical interventions and human trials to exploit, as far as possible, their therapeutic potential for the clinical management of COVID-19 infected patients.
